# Biochemical characterization of FIKK8 – A unique protein kinase from the malaria parasite *Plasmodium falciparum* and other apicomplexans

**DOI:** 10.1016/j.molbiopara.2015.06.002

**Published:** 2015-06

**Authors:** Khan T. Osman, Hua Jane Lou, Wei Qiu, Verena Brand, Aled M. Edwards, Benjamin E. Turk, Raymond Hui

**Affiliations:** a Dept of Molecular Genetics, University of Toronto, Canada; b Structural Genomics Consortium, University of Toronto, Canada; c Dept of Pharmacology, Yale University, United States; d Hospital for Sick Children, Canada; e Toronto General Hospital Research Institute, Canada

**Keywords:** Apicomplexa, FIKK kinase, Kinase, Cryptosporidium

## Abstract

FIKKs are protein kinases with distinctive sequence motifs found exclusively in Apicomplexa. Here, we report on the biochemical characterization of *Plasmodium falciparum* FIKK8 (*Pf*FIKK8) and its *Cryptosporidium parvum* orthologue (*Cp*FIKK) – the only member of the family predicted to be cytosolic and conserved amongst non-*Plasmodium* parasites. Recombinant protein samples of both were catalytically active. We characterized their phosphorylation ability using an enzymatic assay and substrate specificities using an arrayed positional scanning peptide library. Our results show that FIKK8 targets serine, preferably with arginine in the +3 and −3 positions. Furthermore, the soluble and active FIKK constructs in our experiments contained an N-terminal extension (NTE) conserved in FIKK8 orthologues from other apicomplexan species. Based on our results, we propose that this NTE is an integral feature of the FIKK subfamily.

## Highlights

• We studied FIKK kinases from *Plasmodium falciparum* and *Cryptosporidium parvum*.
• Soluble and active samples of PfFIKK8 and CpFIKK contain a N-terminal extension.
• Both FIKK samples preferentially phosphorylated serines with flanking arginines.

## Graphical abstract

**Figure:**
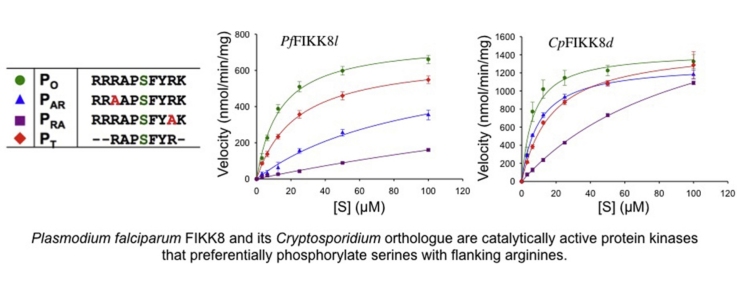

## 1 Introduction

Transmission of malaria, perennially one of the deadliest infectious diseases in the world, is mediated by transmission of *Plasmodium* parasites between a vertebrate host and a mosquito vector. Such a complex life cycle is regulated by a signal transduction system of ∼90 protein kinases (PK) [1], a majority of which having human orthologues. The *Plasmodium* PKs absent from mammalian genomes are likely involved in parasite-specific functions and potential targets of selective drugs. One such group, known as FIKK kinases, is exclusive to the phylum of Apicomplexa and thus, far the subject of few studies.

The defining architecture of the FIKK subfamily includes a highly variable N-terminal region followed by a conserved C-terminal kinase domain (KD) where the eponymous quartet of residues, Phe–Ile–Lys–Lys, is located. Two species, namely *Plasmodium falciparum* and *Plasmodium reichenowi* [2–4] have been reported to have 21 and 6 FIKK members, respectively. Most of these members contain a PEXEL motif – designating export *Plasmodium* proteins [4–7] – as well as a signal peptide in the N-terminus. The sole exception is a single member, orthologous to FIKK8 (*Pf*FIKK8) which is also the longest member. It is also the only member conserved in all *Plasmodium* species, as well as in the genomes of *Toxoplasma*, *Cryptosporidium*, *Neospora* and *Eimeria* [4].

FIKK kinases are expressed mostly during the blood stage of the parasitic life cycle [8]. *Pf*FIKK4.1, *Pf*FIKK9.3, *Pf*FIKK9.6 and *Pf*FIKK12 have been found to be localized in the Maurer’s cleft [8,9]. Furthermore, erythrocytic membrane rigidity has been found to be moderated by gene disruption of either *Pf*FIKK7.1 or *Pf*FIKK12 [10], as well as by dematin, a cytoskeletal protein that is potentially a substrate of *Pf*FIKK4.1 [11]. An attempt to disrupt the FIKK8 orthologue in mouse malaria-causing parasite *Plasmodium berghei* (*Pb*FIKK8) was not successful, suggesting that FIKK8 might play an essential role in cell cycle regulation of *Plasmodium* parasites [12].

Most of the catalytically important residues of typical eukaryotic protein kinases are conserved in FIKK kinases [3], with the exception of the glycine triad, resulting in speculation on their catalytically activity. To date, recombinant samples of FIKK4.1 [11] and FIKK4.2 [9] have been reported to be active. Furthermore, samples of *Pf*FIKK12 and *Pf*FIKK4.1 immuno-precipitated from cell lysates have been found to be capable of phosphorylating myelin basic protein (MBP) [8,11]. In our study, we expressed and purified recombinant protein samples of *P. falciparum* FIKK8 (*Pf*FIKK8, gene ID PF3D7_0805700 at www.plasmodb.org) and its *C. parvum* orthologue *Cp*FIKK (cgd5_4390 at www.cryptodb.org), and studied their enzymatic properties.

## 2 Results

We cloned multiple constructs of *Pf*FIKK8 and *Cp*FIKK (Table S1; method and materials in Supplementary materials). Using an *Escherichia coli* system previously proven for parasite PKs [13], only constructs including an extension of 38 or more residues at the N-terminus of the predicted KD yielded soluble and stable protein samples (Table S1, Fig. S1). This N-terminal extension (NTE) contains both polar and non-polar residues as well as multiple potential phospho-acceptor residues (S/Y) (Fig. S1B). Using a previously described method [14], we purified two constructs of *Pf*FIKK8 and one of *Cp*FIKK containing the NTE (*Pf*FIKK8*l*, *Pf*FIKK8*o* and *Cp*FIKK*d* in Table S1). We then proceeded to assess their auto-phosphorylation behavior and determine their ability to phosphorylate a set of common peptide substrates.

To assess their auto-phosphorylation behavior, the purified *Pf*FIKK8*l* and *Cp*FIKK*d* samples were incubated with ATP and MgCl_2_. The samples were then trypsinized and analyzed using LC–MS–MS. We used the maps of the resulting peptides (Fig. S2) to identify multiple phospho-serines (pS) on the *Plasmodium* sample, both phospho-serines and phospho-threonines (pT) on the *Crytposporidium* sample and one phospho-tyrosine (pY) on each. One of the phosphorylated serines (pS1320) on *Pf*FIKK8*l* is located in the predicted activation loop. Unfortunately, the corresponding serine on *Cp*FIKK*d* was not part of any of the detectable peptides. Furthermore, some of the phosphorylated residues were found in the NTE, including a phospho-serine that is conserved on both samples.

We also found *Pf*FIKK8*l* and *Cp*FIKK*d* to be active against a set of standard kinase substrates – bovine casein, bovine MBP and Syntide-2 (PLARTLSVAGLPGKK), with MBP producing the highest level of activity.

**Fig. 1:**
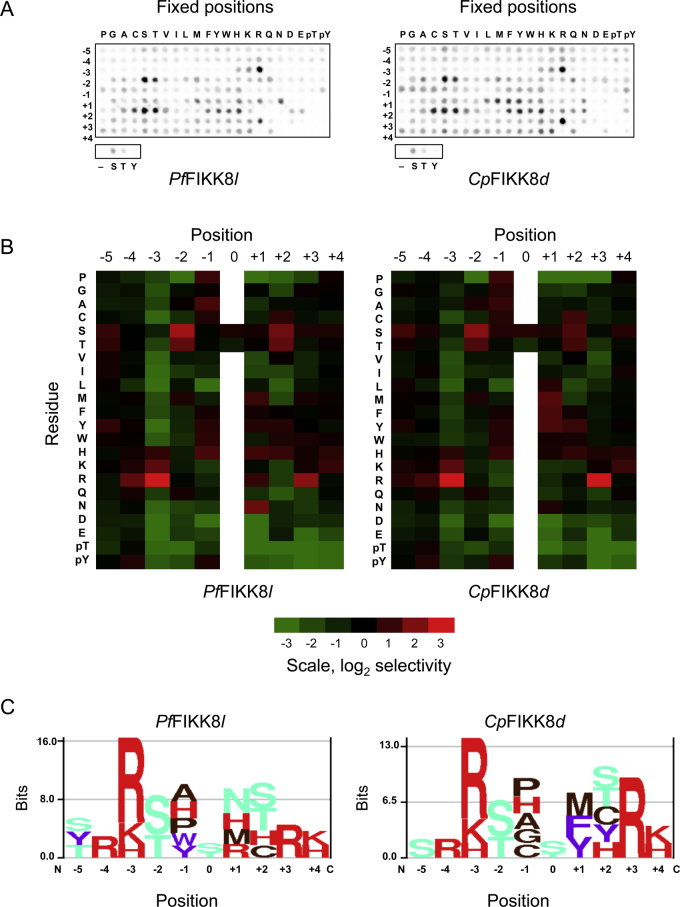
The substrate preferences of *Pf*FIKK8*l* and *Cp*FIKK*d* were assessed using a positional peptide scanning array. (A) A matrix of biotinylated peptides with the indicated residue at the indicated position relative to Ser or Thr was allowed to be phosphorylated by either sample. The reaction mixtures were spotted onto a streptavidin membrane and exposed to a phosphor screen, resulting in a spot image array. (B) The heat maps correspond to normalized signal values in the arrays (average of two runs). Cleary, both samples preferred serine over threonine. (C) Sequence logos illustrate Arg was favored at the –3 and +3 positions by both proteins (preference for Arg at +3 is much stronger for the *Cryptosporidium* sample).

To systematically determine the sequence preferences of *Pf*FIKK8*l* and *Cp*FIKK*d* in an unbiased manner, we assayed the proteins using a positional-scanning peptide array [15]. Both demonstrated a strong preference for basic residues (Figs. 1 and S3 ), primarily Arg at positions −3 and +3 relative to the phosphorylation site. Arginine was also favored by both in the −4 position, albeit not as strongly. In addition, both selected Ser over Thr as the phosphate acceptor by about 2–3-fold (Tables S3 and S4).

**Fig. 2:**
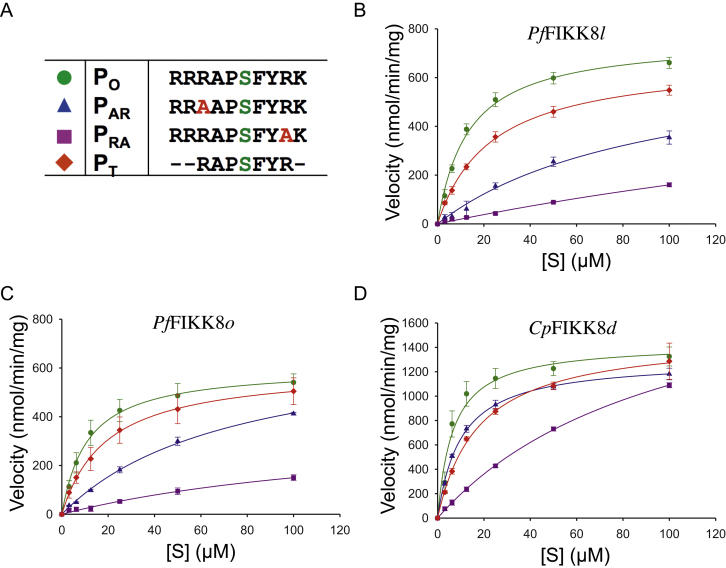
(A) We tested the activity of both *Pf*FIKK8*l* and *Cp*FIKK*d* against four substrates. *P*_O_ featured Arg at −3 and +3 positions. In *P*_AR_ and *P*_RA_, one flanking Arg was replaced in order to assess their importance. *P*_T_ was a truncated form of *P*_O_. (B) *Pf*FIKK8*l* is most active against *P*_O_ and *P*_T_ (the truncated version of *P*_O_). Replacing Arg in the −3 or +3 position (*P*_AR_ and *P*_RA_) substantially reduced activity for the *Plasmodium* sample. (C) While the shorter *Pf*FIKK8o construct was also less active against *P*_AR_ and *P*_RA_, the reduction of activity against *P*_T_ was minimal. (D) A similar trend was seen with the *Cryptosporidium* sample but reduction in activity against the *P*_AR_, *P*_RA_ and *P*_T_ was not as pronounced.

To evaluate the contributions of the arginines and other flanking residues to phosphorylation efficiency, we designed an optimized peptide substrate (*P*_O_) with the sequence RRRAPSFYRK and three variants. The variants featured mutation of Arg to Ala at the +3 (*P*_AR_) and −3 (*P*_RA_), as well as *P*_T_, a truncated version of *P*_O_. Using an LDH–PK coupled kinase assay [16], we compared the phosphorylation kinetics of these substrates with *Pf*FIKK8*l*, *Pf*FIKK8*o* and *Cp*FIKK*d* respectively as catalysts. The two *Pf*FIKK8 constructs behaved very similarly to each other with almost all the peptides (Fig. 2). *Cp*FIKK*d* appeared more active than its *P. falciparum* orthologues; however, all the kinetic parameters are within the same order of magnitude. Similar Michaelis constants (*K*_m_) and catalytic efficiency values (*k*_cat_) were obtained using *P*_O_ and *P*_T_ for all three FIKK samples, with all indicating at least average substrate binding. On the other hand, both mutants (*P*_AR_ and *P*_RA_) resulted in higher *K*_m_ and lower *k*_cat_ values, thus, emphasizing the importance of the flanking arginines.

## 3 Discussion

Many protein kinases, including PKA, PKC and rhoptry kinases (found primarily in *Toxoplasma* [17,18]), have N-terminal and/or C-terminal extensions flanking the canonical bilobed structure. Our results suggest the active domains of *Pf*FIKK8 and *Cp*FIKK have an NTE of at least 38 residues in length. We hypothesize that this extension is an integral component of the kinase domain of both, as neither can be expressed as soluble recombinant proteins in its absence.

In addition to the NTE, the FIKK motif and the absence of a C-terminal extension, FIKK kinases are also defined by a number of conserved divergences in some common kinase motifs. First, Phe and Gly in the DFG triad are often replaced by other hydrophobic residues. Second, in many FIKK kinases, the activation loop features proline in place of the more common alanine in the APE. Furthermore, the HRD motif in subdomain VI features a leucine in place of arginine. The absence of arginine in this position typically signifies that a kinase does not need auto-phosphorylation of the activation loop to become active. Interestingly, we found S1320 in *Pf*FIKK8*l*, which is located in the activation loop and conserved in all FIKK kinases, to be auto-phosphorylated (Fig. S2). To investigate the relevance of this phosphoserine to FIKK8 activation, we attempted to express a mutant form of *Pf*FIKK8*l* with S1320 mutated to alanine; however, the mutated protein did not express. Therefore, a possible regulation mechanism for FIKK kinases mediated by phosphorylation of the activation loop remains to be confirmed.

The inclusion of the NTE as an integral component of *Pf*FIKK8*l* and *Cp*FIKK*d* is corroborated by the kinetic parameters we obtained, all of which are in the range of active protein kinases with average to above average binding affinities for ATP and the optimized substrates used (*P*_O_ and *P*_T_). Furthermore, sequence alignment (not shown) indicates that the NTE is conserved among available FIKK8 orthologues from apicomplexan parasites and, to a lesser degree, the other FIKK paralogues found in *P. falciparum* and *P. reichenowi* (Fig. S4). Given that *Pf*FIKK8*l* and *Pf*FIKK8*o* behaved nearly indistinguishably in the kinetics study, we propose that *Pf*FIKK8*o* specifically defines the boundaries of the active FIKK8 domain and that M1049 is the start of the NTE. Identifying the functional significance of this NTE is left for future research; however, the evidence of auto-phosphorylation discussed above suggests the possibility of a regulatory role.

In addition to phosphoserines in the NTE, our auto-phosphorylation experiment also revealed one phosphorylated site in the N-lobe and 2 more in the C-lobe of *Pf*FIKK8*l*. The N-lobe site, namely S1099, has previously been reported in a phospho-proteomics study of *P. falciparum* [19]. Furthermore, this phosphoserine is conserved in our autophosphorylated *Cp*FIKK*d* sample (Fig. S2) and, significantly, located on the glycine-rich loop of both kinases – implicated in positioning of γ–phosphate in ATP hydrolysis. Previously, a phosphoserine in the same region of yeast ATG1 protein kinase was found to be inhibitory [20].

The peptide array study revealed a preference for Arg at the −3 and +3 positions for *Pf*FIKK8*l* and *Cp*FIKK*d* (Fig. 1) with both enzymes showing the strongest selection for arginine at the +3 position when analyzed using consensus peptide substrates. Notably, the consensus peptides included basic residues at multiple positions upstream of the phosphorylation site (−5, −4 and −3). Therefore, it is possible that the more modest effect of replacing the −3 Arg residue is due to compensation by nearby basic residues. Both *Pf*FIKK8*l* and *Cp*FIKK*d* largely preserved their catalytic efficiencies (based on *k*_cat_/*K*_m_ values in Table S2) when the shortened substrate P_T_ was used in place of P_O_, suggesting that this short substrate may be as an ideal tool for assaying FIKK8 activity and screening for small molecule inhibitors.

In conclusion, our recombinant samples of *Pf*FIKK8 and *Cp*FIKK are orthologous and catalytically active protein kinases, both of which featuring an approximately 40-residue long integral N-terminal extension. Future research to determine the function of this extension may reveal the mechanism of FIKK kinases. It is also possible that, in vivo, regions of the proteins not included in our active constructs may play catalytic, regulatory and localization roles.
