# Supplementary material for: Biochemical characterization of FIKK8 – A unique protein kinase from the malaria parasite Plasmodium falciparum and other apicomplexans
Source: Mol Biochem Parasitol. 2015 Jun;201(2):85–9. doi: 10.1016/j.molbiopara.2015.06.002 (PMC4576209; doi:10.1016/j.molbiopara.2015.06.002)

Supplementary Material:

- Supplementary tables
- Supplementary figures
- Methods and materials

**Table S1**

**Cloning and expression profile of constructs of *Pf*FIKK8 and *Cp*FIKK8.** 21 *Pf*FIKK8 and 6 *Cp*FIKK8 constructs were successfully cloned and tested for expression. The well-expressed constructs (>1 mg soluble and stable protein per litre of culture) are marked by “Y” in the right column. When aligned against known protein kinases with no N-terminal or C-terminal extensions (i.e. kinase domain starting a few residues before the glycine-rich domain), the predicted start of the kinase domains of *Pf*FIKK8 and *Cp*FIKK are positions S1087 and K383 respectively. Amongst the expressing constructs, *Pf*FIKK8*l* (N1025-L1457), *Pf*FIKK8*o* (M1049-L1457) and *Cp*FIKK8*d* (K344-I796) were selected for the biochemical analysis.

| **Annotation** | **FIKK name** | **Start position (aa)** | **Stop position (aa)** | **Protein length (aa)** | **Expression** |
| --- | --- | --- | --- | --- | --- |
| PF3D7_0805700 | *Pf*FIKK8*a* | M1 | L1457 | 1457 | N |
|  | *Pf*FIKK8*b* | D802 | L1457 | 656 | N |
|  | *Pf*FIKK8*c* | N832 | L1457 | 626 | N |
|  | *Pf*FIKK8*d* | P915 | L1457 | 543 | N |
|  | *Pf*FIKK8*e* | S924 | L1457 | 534 | N |
|  | *Pf*FIKK8*f* | L935 | L1457 | 523 | N |
|  | *Pf*FIKK8*g* | M943 | L1457 | 515 | N |
|  | *Pf*FIKK8*h* | K959 | L1457 | 499 | Y |
|  | *Pf*FIKK8*i* | V980 | L1457 | 478 | Y |
|  | *Pf*FIKK8*j* | K990 | L1457 | 468 | Y |
|  | *Pf*FIKK8*k* | Y1002 | L1457 | 456 | Y |
|  | *Pf*FIKK8*l* | N1025 | L1457 | 433 | Y |
|  | *Pf*FIKK8*m* | I1038 | L1457 | 420 | Y |
|  | *Pf*FIKK8*n* | M1041 | L1457 | 417 | Y |
|  | *Pf*FIKK8*o* | M1049 | L1457 | 409 | Y |
|  | *Pf*FIKK8*p* | K1056 | L1457 | 402 | N |
|  | *Pf*FIKK8*q* | S1074 | L1457 | 384 | N |
|  | *Pf*FIKK8*r* | F1080 | L1457 | 378 | N |
|  | *Pf*FIKK8*s* | D1087 | L1457 | 371 | N |
|  | *Pf*FIKK8*t* | T1095 | L1457 | 363 | N |
|  | *Pf*FIKK8*u* | K1100 | L1457 | 358 | N |
| cgd5_4390 | *Cp*FIKK*a* | M1 | I796 | 796 | N |
|  | *Cp*FIKK*b* | E41 | I796 | 756 | N |
|  | *Cp*FIKK*c* | S55 | I796 | 742 | N |
|  | *Cp*FIKK*d* | K344 | I796 | 453 | Y |
|  | *Cp*FIKK*e* | K347 | I796 | 450 | Y |
|  | *Cp*FIKK*f* | N410 | I796 | 387 | N |

Table S2 – List of consensus peptides tested and the kinetic parameters obtained, including the Michaelis constant (K_m_), turnover (k_cat_) and catalytic efficiency (k_cat_/K_m_).

| **Peptides** | | **K_m_ (μM)** | | **k_cat_ (min^-1^)** | | **k_cat_/K_m_ (μM^-1^min^-1^)** | |
| --- | --- | --- | --- | --- | --- | --- | --- |
| Name | Sequence | *Pf*FIKK8*l* | *Cp*FIKK8*d* | *Pf*FIKK8*l* | *Cp*FIKK8*d* | *Pf*FIKK8*l* | *Cp*FIKK8*d* |
| P_O_ | RRRAPSFYRK | 11.0±0.9 | 7.0±1.5 | 31.0±0.7 | 77.0±4.6 | 2.8 | 12 |
| P_AR_ | RRAAPSFYRK | 74.0±6.3 | 10.0±0.4 | 38.0±1.8 | 70.0±0.7 | 0.5 | 7 |
| P_RA_ | RRRAPSFYAK | 150±42 | 100±4 | 20.0±5.6 | 120.0±2.8 | 0.1 | 1.2 |
| P_T_ | --RAPSFYR- | 19.0±0.8 | 18±1 | 31.0±0.5 | 80.0±1.5 | 1.6 | 4.5 |

Table S3 – Kinetic parameters of all three FIKK8 proteins in using ATP (P_T_ was used as the phospho-acceptor substrate).

| **K_m_ (μM)** | | **k_cat_ (min^-1^)** | | **k_cat_/K_m_ (μM^-1^min^-1^)** | |
| --- | --- | --- | --- | --- | --- |
| *Pf*FIKK8*l* | *Cp*FIKK8*d* | *Pf*FIKK8*l* | *Cp*FIKK8*d* | *Pf*FIKK8*l* | *Cp*FIKK8*d* |
| 11.0±0.8 | 48.0±0.9 | 16.0±0.3 | 78.0±0.5 | 1.6 | 1.6 |

Table 4 – Values for *Pf*FIKK8*l* heat map in Fig.1 (average of two runs)

|  | -5 | -4 | -3 | -2 | -1 | 0 | +1 | +2 | +3 | +4 |
| --- | --- | --- | --- | --- | --- | --- | --- | --- | --- | --- |
| P | 0.72 | 0.47 | 0.28 | 0.03 | 2.05 |  | 0.03 | 0.16 | 0.27 | 1.19 |
| G | 0.59 | 0.71 | 0.21 | 0.82 | 1.12 |  | 0.95 | 0.27 | 1.11 | 1.05 |
| A | 0.55 | 0.62 | 0.27 | 1.05 | 2.35 |  | 0.41 | 0.88 | 0.85 | 0.99 |
| C | 0.88 | 0.66 | 0.14 | 0.41 | 1.51 |  | 0.57 | 1.72 | 0.80 | 0.58 |
| S | 1.91 | 1.00 | 0.55 | 5.49 | 0.98 | 1.39 | 1.33 | 3.53 | 1.48 | 1.44 |
| T | 1.74 | 1.00 | 0.28 | 3.10 | 0.96 | 0.61 | 1.05 | 3.08 | 0.71 | 1.09 |
| V | 1.27 | 0.89 | 0.15 | 0.66 | 0.62 |  | 0.25 | 1.14 | 0.50 | 0.86 |
| I | 1.32 | 0.98 | 0.13 | 0.33 | 0.48 |  | 0.28 | 0.26 | 0.51 | 0.99 |
| L | 1.08 | 0.43 | 0.13 | 0.74 | 0.12 |  | 0.69 | 0.18 | 0.67 | 0.91 |
| M | 0.95 | 1.09 | 0.23 | 1.49 | 0.55 |  | 2.05 | 0.24 | 1.35 | 1.13 |
| F | 0.97 | 0.86 | 0.24 | 0.49 | 1.32 |  | 1.26 | 1.09 | 0.98 | 1.10 |
| Y | 1.87 | 1.12 | 0.12 | 0.48 | 1.65 |  | 1.59 | 1.58 | 1.01 | 0.79 |
| W | 1.20 | 1.04 | 0.18 | 0.41 | 1.73 |  | 0.61 | 1.45 | 1.42 | 1.21 |
| H | 0.85 | 1.25 | 1.87 | 0.62 | 2.33 |  | 2.19 | 1.98 | 1.27 | 1.69 |
| K | 1.04 | 1.47 | 2.89 | 0.63 | 0.34 |  | 0.86 | 0.30 | 1.31 | 1.91 |
| R | 0.71 | 2.96 | 11.68 | 0.96 | 0.49 |  | 1.88 | 0.31 | 4.52 | 0.84 |
| Q | 0.71 | 1.32 | 0.31 | 1.34 | 0.68 |  | 0.41 | 0.19 | 0.34 | 0.92 |
| N | 0.58 | 0.93 | 0.15 | 0.28 | 0.33 |  | 3.38 | 0.49 | 0.34 | 0.63 |
| D | 0.57 | 0.52 | 0.07 | 0.32 | 0.12 |  | 0.12 | 0.51 | 0.41 | 0.44 |
| E | 0.48 | 0.67 | 0.12 | 0.34 | 0.28 |  | 0.10 | 0.60 | 0.14 | 0.24 |
| pT | 0.43 | 0.94 | 0.14 | 0.13 | 0.38 |  | 0.05 | 0.11 | 0.06 | 0.14 |
| pY | 0.97 | 1.51 | 0.18 | 0.24 | 1.46 |  | 0.19 | 0.16 | 0.11 | 0.14 |

Table S5 – Values for *Cp*FIKK*d* heat map in Fig. 1 (average of two runs)

|  | -5 | -4 | -3 | -2 | -1 | 0 | +1 | +2 | +3 | +4 |
| --- | --- | --- | --- | --- | --- | --- | --- | --- | --- | --- |
| P | 0.57 | 0.88 | 0.64 | 0.10 | 2.10 |  | 0.07 | 0.09 | 0.11 | 1.17 |
| G | 1.14 | 0.95 | 0.44 | 0.83 | 1.69 |  | 0.37 | 0.30 | 0.67 | 0.75 |
| A | 0.61 | 0.83 | 0.60 | 1.43 | 1.91 |  | 0.64 | 0.74 | 0.48 | 0.97 |
| C | 0.75 | 0.93 | 0.24 | 0.50 | 1.62 |  | 0.65 | 2.14 | 0.53 | 0.71 |
| S | 2.18 | 1.37 | 0.61 | 4.35 | 1.52 | 1.58 | 1.43 | 2.48 | 0.82 | 1.58 |
| T | 1.32 | 0.89 | 0.35 | 2.45 | 0.89 | 0.42 | 0.92 | 2.24 | 0.37 | 0.87 |
| V | 0.75 | 0.96 | 0.22 | 0.68 | 0.68 |  | 0.42 | 1.01 | 0.22 | 0.96 |
| I | 0.69 | 1.11 | 0.23 | 0.45 | 0.53 |  | 0.70 | 0.50 | 0.30 | 1.23 |
| L | 1.24 | 0.60 | 0.17 | 0.82 | 0.19 |  | 1.58 | 0.25 | 0.45 | 0.86 |
| M | 1.08 | 1.02 | 0.35 | 1.43 | 0.62 |  | 2.69 | 0.35 | 0.74 | 0.95 |
| F | 1.05 | 0.79 | 0.29 | 0.76 | 1.27 |  | 2.50 | 1.25 | 0.66 | 0.97 |
| Y | 1.39 | 0.99 | 0.36 | 0.64 | 1.59 |  | 2.44 | 2.04 | 0.73 | 1.07 |
| W | 0.71 | 0.65 | 0.34 | 0.48 | 1.53 |  | 1.33 | 1.35 | 0.72 | 0.87 |
| H | 0.94 | 1.08 | 1.69 | 0.67 | 1.92 |  | 1.36 | 1.80 | 1.46 | 1.65 |
| K | 1.39 | 1.18 | 2.84 | 0.53 | 0.32 |  | 0.37 | 0.42 | 1.18 | 2.04 |
| R | 1.59 | 2.18 | 9.42 | 0.69 | 0.26 |  | 0.46 | 0.82 | 9.37 | 0.91 |
| Q | 0.93 | 1.31 | 0.53 | 1.35 | 0.53 |  | 0.37 | 0.66 | 0.31 | 1.10 |
| N | 0.53 | 0.86 | 0.29 | 0.48 | 0.41 |  | 1.48 | 0.86 | 0.53 | 0.58 |
| D | 0.51 | 0.64 | 0.16 | 0.53 | 0.13 |  | 0.07 | 0.27 | 0.23 | 0.37 |
| E | 0.64 | 0.77 | 0.22 | 0.83 | 0.29 |  | 0.15 | 0.44 | 0.08 | 0.39 |
| pT | 0.87 | 1.24 | 0.32 | 0.23 | 0.43 |  | 0.04 | 0.21 | 0.06 | 0.12 |
| pY | 0.98 | 1.49 | 0.35 | 0.55 | 1.71 |  | 0.34 | 0.49 | 0.06 | 0.16 |

**Methods and Materials**

**Cloning and expression**

The full length DNA constructs of *Pf*FIKK8 and *Cp*FIKK8 were cloned from cDNA library of *P. falciparum* 3D7 (generous donation from the laboratory of Kevin Kain, Toronto General Hospital) and *C. parvum* Iowa I genomic DNA (obtained from MR4) into the pET15-MHL vector (http://www.sgc.utoronto.ca/SGC-WebPages/toronto-vectors.php). DNA constructs were sub-cloned from the full-length constructs and appended with an N-terminal hexa-histidine tag including an integrated TEV cleavage site (MHHHHHHSSGRENLYFQ*G) into the pET15-MHL vector. Small-scale test expression was performed with all the cloned constructs according to Savitsky et al. (2010).

All constructs were grown and purified as previously described [[19](#_ENREF_19)] using the Lex bioreactor system (Harbinger Biotechnology and Engineering Corp., Toronto, ON, Canada) and BL21(DE3)-V2R-pACYC-LamP [[19](#_ENREF_19)] as the expression host, which includes a plasmid for co-expression of λ-phosphatase to suppress protein phosphorylation. To mitigate the effect of the high number of cysteines, 2 mM 2-mercaptoethanol or TCEP was included in every purification steps to minimize aggregation. Both *Pf*FIKK8 and *Cp*FIKK8 kinase domains eluted as monomers from a Superdex S200 gel-filtration column (GE Life Sciences). The identities of the purified proteins were verified by mass spectrometry analysis (ESI-TOF, Agilent Technologies, Toronto, ON, Canada), which also confirmed the absence of phosphorylation. Proteins were concentrated to >8 mg/mL in a buffer containing 10 mM HEPES (pH 7.5), 500 mM NaCl and 2 mM 2-mercaptoethanol or TCEP, and stored at –80^o^C.

**Differential light scattering**

To study the stability of the purified FIKK8 samples and to identify potential ligands, we assayed samples using differential light scattering. This was carried out using the StarGazer instrument (Harbinger Biotechnology and Engineering Corp., Toronto, Canada). The assay was performed using 2 µM of protein samples and 10 mM of ligands in 384-well plates. Samples were buffered in 100 mM HEPES (pH 7.5), 150 mM NaCl. The experiments were conducted between 20°C to 85°C at a heating rate of 1°C per minute. The recorded scattered light reads were fitted to the Boltzmann sigmoid function using Bioactive software and plotted against the temperature (Figure 3), and the inflection point is termed T_m_.

***In vitro* phosphorylation**

For phosphorylation reactions, 20 µM of purified *Pf*FIKK8 and *Cp*FIKK8 were incubated for 12 h, 24 h and 48 h at room temperature in reaction buffer (10 mM HEPES (pH7.5), 500 mM NaCl and 2 mM TCEP) containing 2 mM ATP and 2 mM MgCl_2_, and the reactions were stopped by adding 10 mM EDTA. To measure MBP (Sigma) phosphorylation, 100 µM of MBP was added to 20 µM FIKK8 in reaction buffer containing 2 mM ATP and 2 mM MgCl_2_ and incubated for 12 h at room temperature. Phosphorylation was detected using mass spectrometry, by measuring an 80 Da mass increase using an Agilent electrospray-ionisation time-of-flight (ESI-TOF) mass spectrometer. Prior to injection into the mass spectrometer, the protein was resolved from small molecules by liquid chromatography on a C3 reverse-phase column with 0.1% formic acid and eluted with a methanol gradient. MS data were analyzed with Agilent TOF Protein Confirmation Software.

Phosphorylated residues on FIKK8s and MBP were mapped by LC-MS-MS. For this experiment, proteins were digested with trypsin (13 ng/µL trypsin in 50 mM ammonium bicarbonate and 10 mM DTT) for 3 hours at 37^o^C. The digested peptides were loaded onto a 150 μm ID pre-column (Magic C18, Michrom Biosciences) at 4 μL/min and separated over a 75 μm ID analytical column packed into an emitter tip containing the same packing material. The peptides were eluted over 60 min. at 300 nL/min. using a 0 to 40% acetonitrile gradient in 0.1% formic acid using an EASY n-LC nano-chromatography pump (Proxeon Biosystems, Odense Denmark). The peptides were eluted into an LTQ-Orbitrap hybrid mass spectrometer (Thermo-Fisher, Bremen, Germany) operated in a data dependent mode. MS was acquired at 60,000 FWHM resolutions in the FTMS and MS-MS was carried out in the linear ion trap. 6 MS-MS scans were obtained per MS cycle. The Raw data was searched using Mascot (Matrix Sciences, London UK). The final data were analyzed with SF3 software (Proteome Software, Scaffold, http://www.proteomesoftware.com/products/scaffold)

**Kinase substrate peptide array**

The substrate specificities of *Pf*FIKKs and *Cp*FIKK were determined by incubating 20 nM of each purified enzyme with an array of 200 peptide mixtures (50 µM in 50 mM HEPES, pH 7.4, 150 mM NaCl, 5 mM MgCl_2_, 1 mM DTT, 0.1% Tween 20, 50 µM ATP including 0.03 µCi/µl [γ-^33^P]ATP) as described [[20](#_ENREF_20)]. Most (198) peptide mixtures had the general sequence Y-A-X-X-X-X-X-S/T-X-X-X-X-A-K-K(biotin), where X indicates an equimolar mixture of the 17 amino acids excluding Cys, Ser and Thr, and S/T indicates an equimolar mixture of Ser and Thr. In the context of this sequence, a single X position was fixed as one of the 20 unmodified amino acids, pThr or pTyr. Two additional peptides having the sequence Y-A-X-X-X-X-X-Z-X-X-X-X-A-K-K(biotin), in which Z was either Ser or Thr, were included to assess phosphoacceptor residue preference. The assay was performed in 1536-well plates (2 µl/well). After incubating 2 hr at 30 ºC, aliquots (200 nl) from each well were spotted onto a streptavidin membrane (SAM2 biotin capture membrane, Promega), which was washed, dried and exposed to a phosphor imager screen. Spot intensities were quantified using the software accompanying the imaging system (QuantityOne, BioRad). The intensity data were normalized by dividing each value by the average of all values corresponding to a single position in the peptide. Heat maps were generated from log transformed quantified data (average of two separate experiments for each kinase) using Microsoft Excel.

**Enzymatic assay**

Kinase activity was measured using an NADH-coupled LDH-PK ATPase assay according to the method of previously described by Kiianitsa et al. (2003). A 96-well format was used and the method was adapted for use with high ATP concentrations. Peptides were obtained from Peptide 2.0 (Peptide 2.0 Inc., http://www.peptide2.com). The proteins at different concentrations (1 nM, 5 nM and 10 nM) for different peptides were pre-incubated in reaction solution (including 0.5 mM ATP, 150 µM NADH, 30 µM PEP, LDH-PK mix from Sigma (with 3 units of LDH per ml) and 10 mM MgCl_2_) for 30 mins at room temperature. A 2 fold serial dilution of peptides starting from 100 µM was added to initiate the reaction. For measuring K_m_ of ATP, 50 µM of P_T_ (RAPSFYR) was used as phospho-acceptor substrate; and ATP was diluted serially from 250 µM (2 fold serial dilution). In this case, the reaction was initiated by adding ATP to the reaction solution. Initial rates were calculated and at least triplicate data were analyzed using SigmaPlot 9 (Systat Software Inc., <http://www.sigmaplot.com>).

**Fig. S1.** (A) Test expression gel showing that constructs *Pf*FIKK8*h* to *Pf*FIKK8*o* (see Table S1) were soluble. (B) The shortest soluble construct was *Pf*FIKK8*o*, containing a N-terminal extension starting at S1049. The predicted kinase domain based on alignment with known kinases without an N-terminal extension (i.e. a few amino acids upstream of the glycline-rich region) starts around *Pf*FIKK8*s* at D1087.


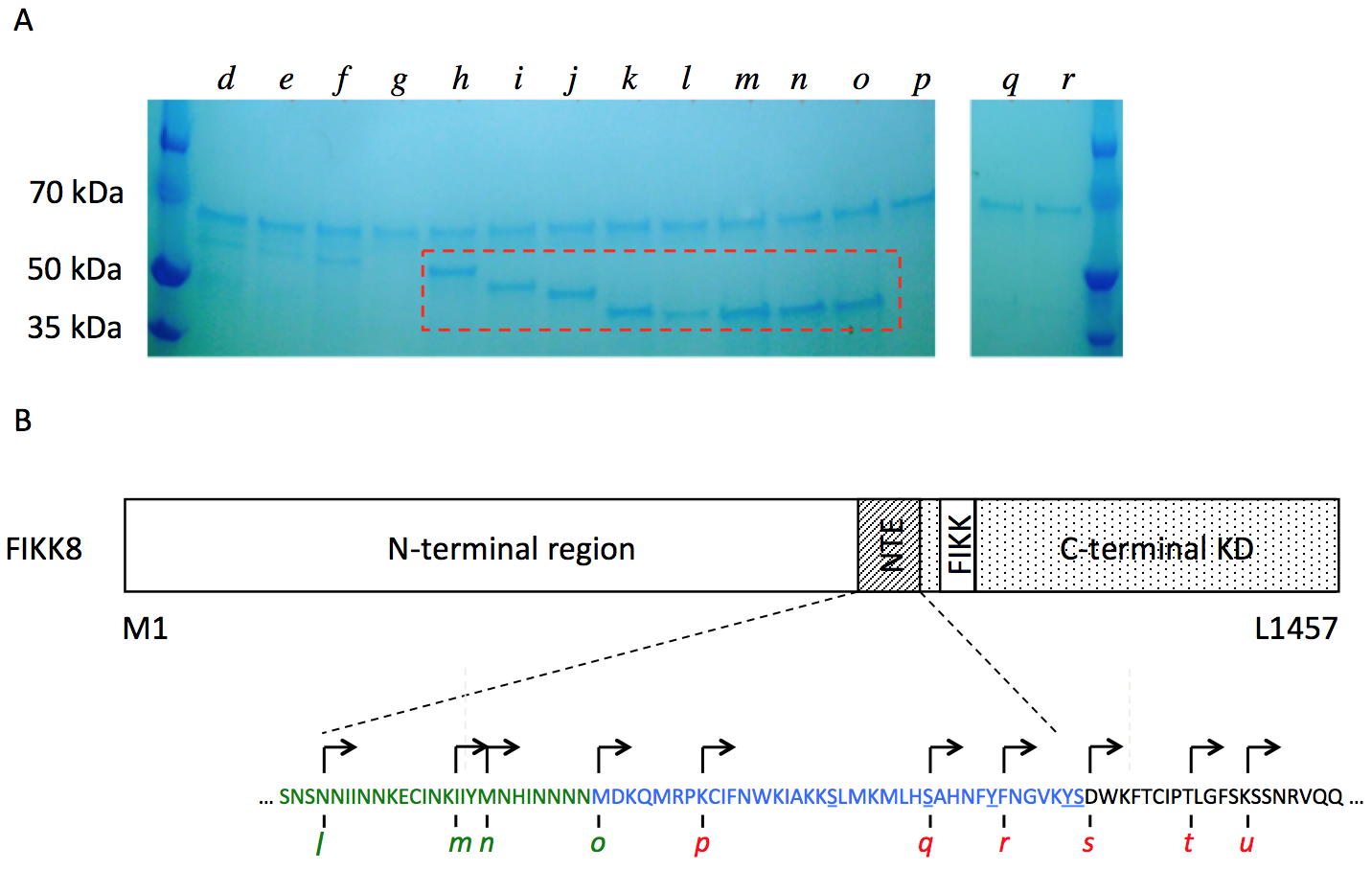


**Fig. S2.** Maps of phosphorylated residues in (A) *Pf*FIKK8*l* and (B) *Cp*FIKK*d*. Residues with post-translational modifications are highlighted in green. Yellow regions represented those included in MS analysis. Phospho-serines in the NTE are highlighted in red while those in the catalytic domain are circled in blue. Conserved phosphoserines are highlighted in solid squares. Serines that are conserved in both proteins but only phosphorylated in one are highlighted in squares with a dashed border. The latter includes a serine in the activation loop (pS1320) on *Pf*FIKK8*l*. While this serine is conserved in *Cp*FIKK*d*, it was not covered in the MS analysis.

A


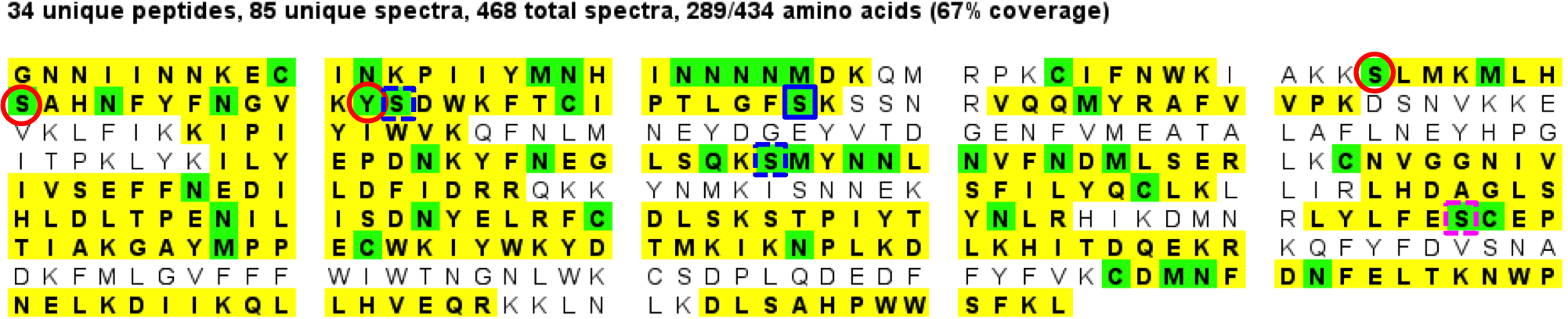


B


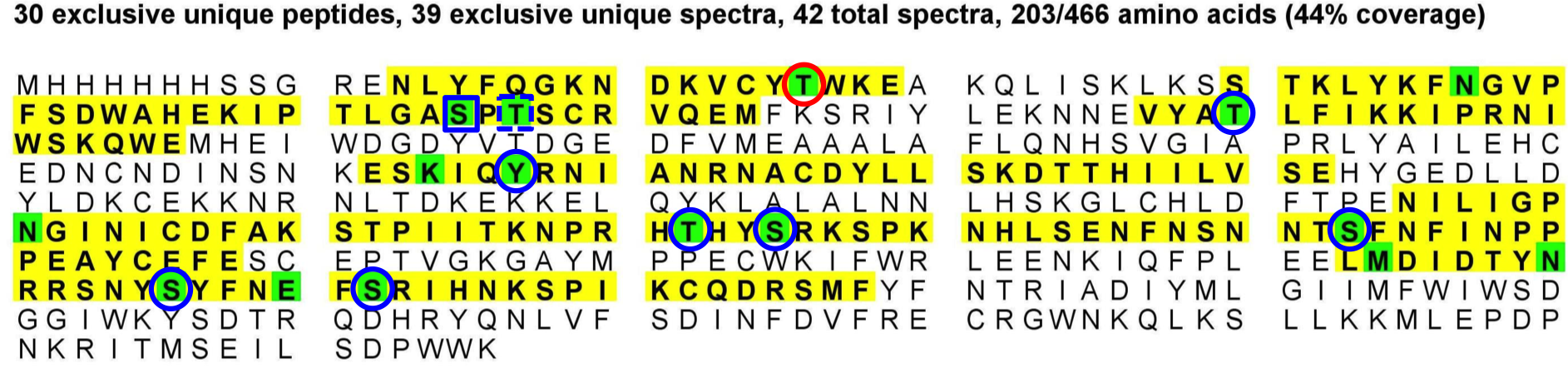


**Fig. S3.** Spot array images for *Pf*FIKK8*l* and *Cp*FIKK*d*.


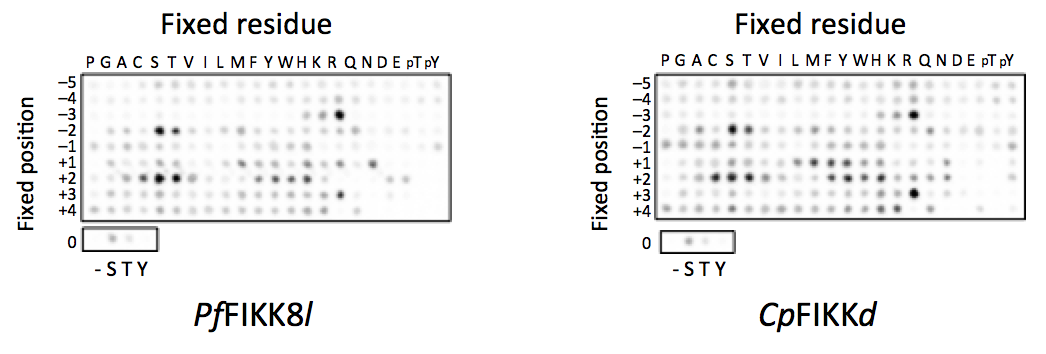


**Fig. S4.** Sequence alignment showing conserved residues in the putative NTE regions of *Plasmodium falciparum* FIKK kinases. Two tryptophans and a glycine, outlined in red, are conserved across the whole family. In addition, an LKM motif (in green) and an NFSINGVKYDDW motif (in magenta) are conserved amongst at least half of the members.


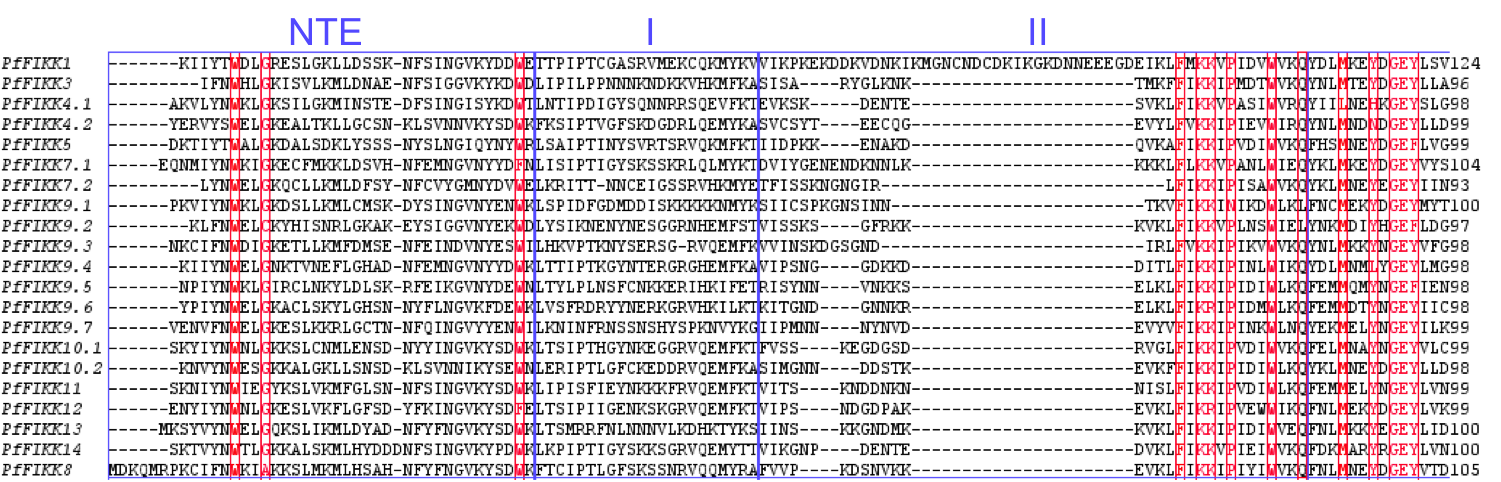

Supplement: Supplementary file 1 [file mmc1.docx]
